# 1-(3-Hy­droxy­phen­yl)-3-(3-meth­oxy­phenyl)thio­urea

**DOI:** 10.1107/S1600536812002553

**Published:** 2012-01-31

**Authors:** Hyeong Choi, Yong Suk Shim, Byung Hee Han, Sung Kwon Kang, Chang Keun Sung

**Affiliations:** a Department of Chemistry, Chungnam National University, Daejeon 305-764, Republic of Korea; b Department of Food Science and Technology, Chungnam National University, Daejeon 305-764, Republic of Korea

## Abstract

In the title compound, C_14_H_14_N_2_O_2_S, the dihedral angles between the thio­urea group and the methoxyphenyl and hydroxyphenyl rings are 61.91 (4) and 76.90 (4)°, respectively. The benzene rings are twisted with respect to each other, making a dihedral angle of 71.03 (4)°. The H atoms of the thio­urea NH groups are positioned *anti* to each other. In the crystal, inter­molecular N—H⋯S, N—H⋯O and O—H⋯S hydrogen bonds link the mol­ecules into a three-dimensional network.

## Related literature

For general background to tyrosinase, see: Kubo *et al.* (2000 ▶). For the development of tyrosinase inhibitors, see: Son *et al.* (2000 ▶); Iida *et al.* (1995 ▶); Kojima *et al.* (1995 ▶); Cabanes *et al.* (1994 ▶).
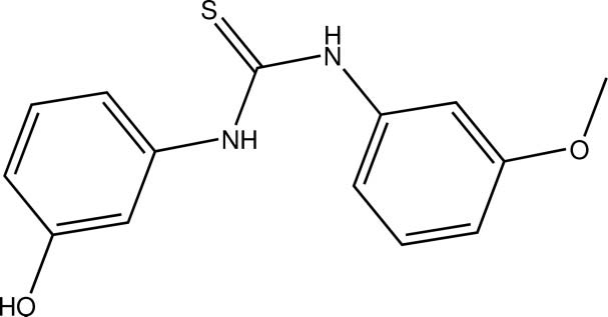


## Experimental

### 

#### Crystal data

C_14_H_14_N_2_O_2_S
*M* *_r_* = 274.33
Triclinic, 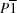

*a* = 6.9925 (4) Å
*b* = 9.8666 (6) Å
*c* = 10.4238 (6) Å
α = 103.055 (2)°
β = 100.033 (1)°
γ = 90.508 (1)°
*V* = 688.99 (7) Å^3^
*Z* = 2
Mo *K*α radiation
μ = 0.23 mm^−1^
*T* = 296 K
0.2 × 0.17 × 0.08 mm

#### Data collection

Bruker SMART CCD area-detector diffractometer
Absorption correction: multi-scan (*SADABS*; Bruker, 2002 ▶) *T*_min_ = 0.956, *T*_max_ = 0.975
26807 measured reflections
3419 independent reflections
2503 reflections with *I* > 2σ(*I*)
*R*_int_ = 0.046

#### Refinement

*R*[*F* ^2^ > 2σ(*F* ^2^)] = 0.036
*wR*(*F* ^2^) = 0.097
*S* = 1.01
3419 reflections
184 parameters
H atoms treated by a mixture of independent and constrained refinement
Δρ_max_ = 0.18 e Å^−3^
Δρ_min_ = −0.20 e Å^−3^

### 

Data collection: *SMART* (Bruker, 2002 ▶); cell refinement: *SAINT* (Bruker, 2002 ▶); data reduction: *SAINT*; program(s) used to solve structure: *SHELXS97* (Sheldrick, 2008 ▶); program(s) used to refine structure: *SHELXL97* (Sheldrick, 2008 ▶); molecular graphics: *ORTEP-3 for Windows* (Farrugia, 1997 ▶); software used to prepare material for publication: *WinGX* (Farrugia, 1999 ▶).

## Supplementary Material

Crystal structure: contains datablock(s) global, I. DOI: 10.1107/S1600536812002553/bh2409sup1.cif

Structure factors: contains datablock(s) I. DOI: 10.1107/S1600536812002553/bh2409Isup2.hkl

Supplementary material file. DOI: 10.1107/S1600536812002553/bh2409Isup3.cml

Additional supplementary materials:  crystallographic information; 3D view; checkCIF report

## Figures and Tables

**Table 1:** Hydrogen-bond geometry (Å, °)

*D*—H⋯*A*	*D*—H	H⋯*A*	*D*⋯*A*	*D*—H⋯*A*
N7—H7⋯S9^i^	0.832 (16)	2.588 (16)	3.3683 (12)	156.8 (14)
N10—H10⋯O17^ii^	0.807 (16)	2.239 (16)	2.9547 (16)	148.0 (15)
O19—H19⋯S9^iii^	0.90 (2)	2.35 (3)	3.2424 (12)	170 (2)

Symmetry codes: (i) 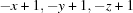; (ii) 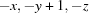; (iii) 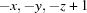.

**Fig. 1.**
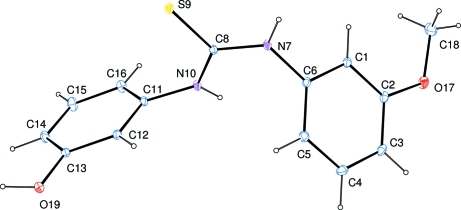
Molecular structure of the title compound, showing the atom-numbering scheme and 30% probability ellipsoids.

**Fig. 2.**
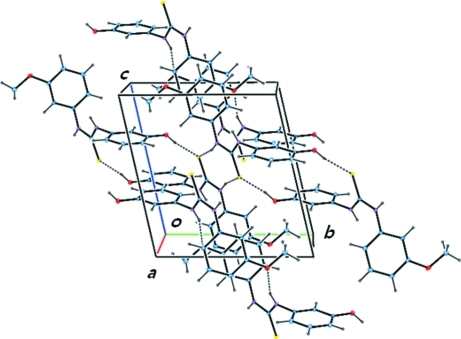
Part of the packing diagram of the title compound, showing a three-dimensional network of molecules linked by intermolecular N—H···O, N—H···S and O—H···S hydrogen bonds (dashed lines).

## supplementary crystallographic information

### Comment

Tyrosinase, a multifunctional copper-containing enzyme, is widely distributed in nature. It is the key enzyme in the undesirable browning of fruits and vegetables, and coloring of skin, hair, and eyes in animals (Kubo *et al.*, 2000). Its inhibition is one of the major strategies in developing new whitening agents. Numerous potential tyrosinase inhibitors have been discovered from natural and synthetic sources, such as ascorbic acid (Kojima *et al.*, 1995), kojic acid (Cabanes *et al.*, 1994), and tropolone (Son *et al.*, 2000; Iida *et al.*, 1995). But some of their individual activities are either not potent enough to be considered of practical use or not compatible with safety regulations for food and cosmetic additives. In our continuing search for tyrosinase inhibitors, we have synthesized the title compound from the reaction of 3-methoxyphenyl-isothiocyanate and 3-aminophenol under ambient conditions. Here, its structure is described (Fig. 1).

The 3-methoxyphenyl and 3-hydroxyphenyl moieties are almost planar with r.m.s. deviations of 0.008 and 0.016 Å, respectively, from the corresponding least-squares planes defined by the eight constituent atoms. The dihedral angles between thiourea moiety (N7···N10) and two benzene groups, C1···N7 and N10···C16, are 61.91 (4) and 76.90 (4)°, respectively. And two benzene groups are twisted to each other with the dihedral angle of 71.03 (4)°. The H7 and H10 atoms of the thiourea NH groups are positioned *anti* to each other (Fig. 1). The presence of intermolecular N7—H7···S9^i^, N10—H10···O17^ii^ and O19—H19···S9^iii^ [symmetry codes: (i) -*x*+1, -*y*+1, -*z*+1, (ii) -*x*, -*y*+1, -*z*, (iii) -*x*, -*y*, -*z*+1] hydrogen bonds link the molecules into a three-dimensional network (Fig. 2 and Table 1). The thiourea-S9 accepts two hydrogen bonds, each from NH and OH groups.

### Experimental

3-Methoxyphenyl isothiocyanate and 3-aminophenol were purchased from Sigma Chemical Co. Solvents used for organic synthesis were distilled before use. All other chemicals and solvents were of analytical grade and were used without further purification. The title compound was prepared from the reaction of 3-methoxyphenyl isothiocyanate (0.2 g, 1.0 mmol) with 3-aminophenol (0.2 g, 1.2 mmol) in acetonitrile (6 ml) under stirring. The reaction was completed within 1 h at room temperature. The solvent was removed under reduced pressure and the product washed with dichloromethane. Removal of the solvent gave a white solid (91%; m.p. 401 K). Colourless crystals were obtained by slow evaporation of an ethanol solution at room temperature.

### Refinement

H atoms of the NH and OH groups were located in a difference Fourier map and refined freely [refined distances; N—H = 0.81 (2)-0.83 (2) Å, O—H = 0.90 (2) Å]. Other H atoms were positioned geometrically and refined using a riding model, with C—H = 0.93 or 0.96 Å, and with *U*_iso_(H) = 1.2*U*_eq_(carrier C) for aromatic or 1.5*U*_eq_(carrier C) for methyl H atoms

### Crystal data

**Table:**

C_14_H_14_N_2_O_2_S	*Z* = 2
*M**_r_* = 274.33	*F*(000) = 288
Triclinic, *P*1	*D*_x_ = 1.322 Mg m^−^^3^
Hall symbol: -P 1	Melting point: 401 K
*a* = 6.9925 (4) Å	Mo *K*α radiation, λ = 0.71073 Å
*b* = 9.8666 (6) Å	Cell parameters from 5768 reflections
*c* = 10.4238 (6) Å	θ = 2.6–27.4°
α = 103.055 (2)°	µ = 0.23 mm^−^^1^
β = 100.033 (1)°	*T* = 296 K
γ = 90.508 (1)°	Block, colourless
*V* = 688.99 (7) Å^3^	0.2 × 0.17 × 0.08 mm

### Data collection

**Table:**

Bruker SMART CCD area-detector diffractometer	2503 reflections with *I* > 2σ(*I*)
graphite	*R*_int_ = 0.046
φ and ω scans	θ_max_ = 28.3°, θ_min_ = 2.0°
Absorption correction: multi-scan (*SADABS*; Bruker, 2002)	*h* = −9→9
*T*_min_ = 0.956, *T*_max_ = 0.975	*k* = −13→13
26807 measured reflections	*l* = −13→13
3419 independent reflections	

### Refinement

**Table:**

Refinement on *F*^2^	Primary atom site location: structure-invariant direct methods
Least-squares matrix: full	Secondary atom site location: difference Fourier map
*R*[*F*^2^ > 2σ(*F*^2^)] = 0.036	Hydrogen site location: inferred from neighbouring sites
*wR*(*F*^2^) = 0.097	H atoms treated by a mixture of independent and constrained refinement
*S* = 1.01	*w* = 1/[σ^2^(*F*_o_^2^) + (0.0503*P*)^2^] where *P* = (*F*_o_^2^ + 2*F*_c_^2^)/3
3419 reflections	(Δ/σ)_max_ < 0.001
184 parameters	Δρ_max_ = 0.18 e Å^−^^3^
0 restraints	Δρ_min_ = −0.20 e Å^−^^3^
0 constraints	

### Fractional atomic coordinates and isotropic or equivalent isotropic displacement parameters (Å^2^)

**Table:**

	*x*	*y*	*z*	*U*_iso_*/*U*_eq_	
C1	0.24836 (18)	0.58574 (16)	0.14956 (13)	0.0403 (3)	
H1	0.2357	0.6671	0.2123	0.048*	
C2	0.23577 (19)	0.58682 (17)	0.01519 (14)	0.0438 (4)	
C3	0.2516 (2)	0.46570 (19)	−0.07750 (15)	0.0501 (4)	
H3	0.2405	0.4666	−0.1675	0.06*	
C4	0.2838 (2)	0.34340 (19)	−0.03709 (16)	0.0544 (4)	
H4	0.2945	0.2617	−0.1001	0.065*	
C5	0.3005 (2)	0.34057 (17)	0.09647 (16)	0.0491 (4)	
H5	0.3253	0.258	0.1239	0.059*	
C6	0.28010 (19)	0.46141 (16)	0.18824 (13)	0.0395 (3)	
N7	0.29788 (18)	0.46161 (13)	0.32718 (12)	0.0439 (3)	
H7	0.387 (2)	0.5124 (16)	0.3796 (16)	0.053 (5)*	
C8	0.19730 (19)	0.37739 (14)	0.37909 (13)	0.0359 (3)	
S9	0.26879 (6)	0.36711 (4)	0.53992 (4)	0.04690 (14)	
N10	0.04156 (17)	0.30682 (13)	0.29859 (12)	0.0412 (3)	
H10	0.007 (2)	0.3295 (17)	0.2288 (16)	0.050 (5)*	
C11	−0.07082 (19)	0.19821 (14)	0.32679 (13)	0.0376 (3)	
C12	0.0066 (2)	0.07050 (14)	0.32391 (13)	0.0397 (3)	
H12	0.1324	0.0564	0.3077	0.048*	
C13	−0.1033 (2)	−0.03815 (15)	0.34530 (13)	0.0407 (3)	
C14	−0.2904 (2)	−0.01681 (17)	0.36628 (16)	0.0530 (4)	
H14	−0.366	−0.0894	0.3783	0.064*	
C15	−0.3659 (2)	0.11175 (19)	0.3695 (2)	0.0643 (5)	
H15	−0.4917	0.1257	0.3858	0.077*	
C16	−0.2575 (2)	0.22180 (17)	0.34868 (17)	0.0542 (4)	
H16	−0.3097	0.3083	0.3496	0.065*	
O17	0.20472 (17)	0.70305 (13)	−0.03543 (11)	0.0593 (3)	
C18	0.2042 (3)	0.8340 (2)	0.0567 (2)	0.0800 (6)	
H18A	0.1815	0.9061	0.0082	0.12*	
H18B	0.3277	0.8528	0.116	0.12*	
H18C	0.1031	0.8313	0.1078	0.12*	
O19	−0.01696 (17)	−0.16246 (11)	0.34188 (11)	0.0543 (3)	
H19	−0.080 (3)	−0.213 (3)	0.385 (2)	0.110 (8)*	

### Atomic displacement parameters (Å^2^)

**Table:**

	*U*^11^	*U*^22^	*U*^33^	*U*^12^	*U*^13^	*U*^23^
C1	0.0375 (7)	0.0498 (9)	0.0336 (7)	−0.0083 (6)	0.0027 (6)	0.0126 (6)
C2	0.0341 (7)	0.0620 (10)	0.0381 (8)	−0.0061 (6)	0.0013 (6)	0.0216 (7)
C3	0.0393 (8)	0.0787 (12)	0.0317 (8)	−0.0072 (7)	0.0041 (6)	0.0136 (8)
C4	0.0491 (9)	0.0648 (11)	0.0425 (9)	−0.0062 (8)	0.0096 (7)	−0.0022 (8)
C5	0.0487 (8)	0.0509 (9)	0.0470 (9)	−0.0063 (7)	0.0066 (7)	0.0121 (8)
C6	0.0344 (7)	0.0515 (9)	0.0319 (7)	−0.0131 (6)	0.0010 (5)	0.0127 (6)
N7	0.0487 (7)	0.0492 (8)	0.0318 (6)	−0.0212 (6)	−0.0042 (5)	0.0148 (6)
C8	0.0411 (7)	0.0333 (7)	0.0328 (7)	−0.0049 (6)	0.0015 (6)	0.0108 (6)
S9	0.0577 (2)	0.0490 (2)	0.0323 (2)	−0.02053 (17)	−0.00393 (16)	0.01601 (16)
N10	0.0430 (6)	0.0451 (7)	0.0356 (7)	−0.0136 (5)	−0.0051 (5)	0.0197 (6)
C11	0.0402 (7)	0.0392 (8)	0.0317 (7)	−0.0112 (6)	−0.0015 (6)	0.0116 (6)
C12	0.0420 (7)	0.0418 (8)	0.0348 (7)	−0.0067 (6)	0.0060 (6)	0.0092 (6)
C13	0.0525 (8)	0.0371 (8)	0.0303 (7)	−0.0087 (6)	0.0023 (6)	0.0077 (6)
C14	0.0492 (9)	0.0516 (10)	0.0594 (10)	−0.0168 (7)	0.0060 (7)	0.0193 (8)
C15	0.0391 (8)	0.0711 (12)	0.0908 (14)	−0.0043 (8)	0.0140 (8)	0.0339 (11)
C16	0.0448 (8)	0.0510 (10)	0.0710 (11)	0.0011 (7)	0.0076 (8)	0.0249 (8)
O17	0.0678 (7)	0.0717 (8)	0.0435 (6)	0.0008 (6)	0.0024 (5)	0.0301 (6)
C18	0.1086 (16)	0.0616 (13)	0.0721 (14)	−0.0002 (11)	0.0033 (12)	0.0304 (11)
O19	0.0752 (8)	0.0382 (6)	0.0528 (7)	−0.0032 (5)	0.0191 (6)	0.0121 (5)

### Geometric parameters (Å, °)

**Table:**

C1—C6	1.3834 (19)	N10—H10	0.807 (16)
C1—C2	1.3904 (18)	C11—C12	1.3721 (19)
C1—H1	0.93	C11—C16	1.376 (2)
C2—O17	1.3706 (18)	C12—C13	1.3938 (18)
C2—C3	1.376 (2)	C12—H12	0.93
C3—C4	1.373 (2)	C13—O19	1.3677 (17)
C3—H3	0.93	C13—C14	1.374 (2)
C4—C5	1.383 (2)	C14—C15	1.374 (2)
C4—H4	0.93	C14—H14	0.93
C5—C6	1.376 (2)	C15—C16	1.397 (2)
C5—H5	0.93	C15—H15	0.93
C6—N7	1.4314 (17)	C16—H16	0.93
N7—C8	1.3429 (17)	O17—C18	1.425 (2)
N7—H7	0.832 (16)	C18—H18A	0.96
C8—N10	1.3338 (16)	C18—H18B	0.96
C8—S9	1.6900 (13)	C18—H18C	0.96
N10—C11	1.4353 (16)	O19—H19	0.90 (2)
			
C6—C1—C2	118.81 (14)	C12—C11—C16	121.41 (13)
C6—C1—H1	120.6	C12—C11—N10	119.07 (12)
C2—C1—H1	120.6	C16—C11—N10	119.42 (13)
O17—C2—C3	115.33 (13)	C11—C12—C13	119.98 (13)
O17—C2—C1	124.32 (15)	C11—C12—H12	120
C3—C2—C1	120.35 (14)	C13—C12—H12	120
C4—C3—C2	119.98 (14)	O19—C13—C14	123.60 (13)
C4—C3—H3	120	O19—C13—C12	117.00 (13)
C2—C3—H3	120	C14—C13—C12	119.40 (14)
C3—C4—C5	120.59 (15)	C15—C14—C13	120.02 (14)
C3—C4—H4	119.7	C15—C14—H14	120
C5—C4—H4	119.7	C13—C14—H14	120
C6—C5—C4	119.14 (15)	C14—C15—C16	121.26 (15)
C6—C5—H5	120.4	C14—C15—H15	119.4
C4—C5—H5	120.4	C16—C15—H15	119.4
C5—C6—C1	121.10 (13)	C11—C16—C15	117.91 (15)
C5—C6—N7	120.30 (14)	C11—C16—H16	121
C1—C6—N7	118.56 (13)	C15—C16—H16	121
C8—N7—C6	126.11 (12)	C2—O17—C18	118.09 (13)
C8—N7—H7	116.8 (11)	O17—C18—H18A	109.5
C6—N7—H7	116.7 (11)	O17—C18—H18B	109.5
N10—C8—N7	116.72 (12)	H18A—C18—H18B	109.5
N10—C8—S9	123.46 (10)	O17—C18—H18C	109.5
N7—C8—S9	119.81 (10)	H18A—C18—H18C	109.5
C8—N10—C11	125.96 (12)	H18B—C18—H18C	109.5
C8—N10—H10	116.2 (11)	C13—O19—H19	108.9 (15)
C11—N10—H10	117.9 (11)		

### Hydrogen-bond geometry (Å, °)

**Table:**

*D*—H···*A*	*D*—H	H···*A*	*D*···*A*	*D*—H···*A*
N7—H7···S9^i^	0.832 (16)	2.588 (16)	3.3683 (12)	156.8 (14)
N10—H10···O17^ii^	0.807 (16)	2.239 (16)	2.9547 (16)	148.0 (15)
O19—H19···S9^iii^	0.90 (2)	2.35 (3)	3.2424 (12)	170 (2)

Symmetry codes: (i) −*x*+1, −*y*+1, −*z*+1; (ii) −*x*, −*y*+1, −*z*; (iii) −*x*, −*y*, −*z*+1.
